# Industry Payments and Sentiments Toward Robotic Surgery Among US Physicians

**DOI:** 10.1001/jamanetworkopen.2024.58552

**Published:** 2025-02-07

**Authors:** Wei San Loh, Andrew M. Ibrahim, Sarah Sheskey, Colleen M. Stone, Kyle H. Sheetz

**Affiliations:** 1 Center for Healthcare Outcomes & Policy, University of Michigan, Ann Arbor; 2 Department of Surgery, University of Michigan, Ann Arbor; 3 Taubman College of Architecture & Urban Planning, University of Michigan, Ann Arbor

## Abstract

**Importance:**

Given the rapid adoption of robotic surgery and its association with substantial industry payments, objective documentation of physicians’ perceptions toward robotic surgery is important.

**Objective:**

To assess US-based physicians’ sentiments toward robotic surgery and whether industry payments are associated with their perceptions.

**Design, Setting, and Participants:**

This cohort study analyzed publicly available posts made from March 19, 2009, to April 1, 2024, by 268 US-based physicians who followed the Society of American Gastrointestinal and Endoscopic Surgeons on X (formerly Twitter). Trainees and individuals without validated clinical practices were excluded. Physicians’ specialties were confirmed using the National Provider Identifier Registry.

**Exposure:**

Payments from Intuitive Surgical Inc, a biotechnology company that manufactures robotic products for use in surgery, were identified via the Open Payments website. Among the physicians, 177 (66.0%) received payments and 91 (34.0%) did not.

**Main Outcomes and Measures:**

Polarity and subjectivity scores of social media posts shared by physicians. Polarity was scored from −1 to 1, with higher scores reflecting more positive sentiment, lower scores reflecting more negative sentiment, and 0 indicating neutrality. Subjectivity was scored from 0 to 1, with higher scores reflecting more subjective opinions and lower scores reflecting objective facts.

**Results:**

This study comprised 268 physicians (154 men [57.5%], 68 women [25.4%], and 46 physicians [17.2%] with sex not reported; 113 general surgeons [42.2%] and 155 physicians in surgical subspecialties [57.8%]). The overall sentiment scores ranged from −0.25 to 0.5 (mean [SD] score, 0.1 [0.2]; 95% CI, 0.09-0.13) and subjectivity scores ranged from 0 to 0.9 (mean [SD] score, 0.4 [0.2]; 95% CI, 0.38-0.41), suggesting that posts were relatively objective with occasional personal insights. No significant difference was seen in mean polarity scores between the 177 physicians who received payments and the 91 physicians who did not (mean [SD] score, 0.12 [0.2]; 95% CI, 0.09-0.14 vs 0.1 [0.2]; 95% CI, 0.07-0.14). Physicians posting before and after receiving industry payments showed consistent positive sentiments (median polarity, 0.1 [IQR, 0.03-0.21]) and decreased subjectivity after receiving payment. Physicians in the top 25% of payment distribution had more positive sentiments and increased subjectivity after receiving payments, whereas those in the bottom 75% showed little change.

**Conclusions and Relevance:**

In this cohort study of US-based physicians, publicly available social media data were used to quantify perceptions of robotic surgery. These findings demonstrate the potential of such data to inform health care practices, guide balanced information dissemination, and uphold the integrity of health information shared by physicians.

## Key Points

**Question:**

How do US-based physicians perceive robotic surgery, and are their perceptions associated with industry payments?

**Findings:**

In this cohort study using publicly available social media posts by 268 physicians, sentiment scores were similar between those who received payments and those who did not. Physicians in the top 25% of payment distribution had more positive sentiments about robotic surgery after receiving payments, whereas no change was observed for the bottom 75%.

**Meaning:**

This study demonstrates the potential of using publicly available social media data to objectively understand professional discourse in the medical field, highlighting the possible influence of industry payments on physician sentiments.

## Teaser

This cohort study uses publicly available social media posts to assess US-based physicians’ sentiments toward robotic surgery and whether industry payments are associated with those perceptions.

## Introduction

Robotic surgery has seen a significant increase in adoption across various general surgery subspecialties. From 2008 to 2017, the annual volume of robotic procedures increased more than 6-fold, and the proportion of surgeons performing robotic surgery quadrupled from 2012 to 2018.^1,2^ Despite this rapid adoption, a gap remains in understanding physicians’ sentiments toward robotic surgery within these subspecialities. Although existing literature has explored the adoption and clinical outcomes of robotic surgery,^3,4,5^ it has largely overlooked the informal yet important sentiments expressed by physicians. Understanding these perceptions is crucial, as physicians’ opinions often shape public health policies and implementation strategies in medical practice.

This study aims to formally document and characterize physicians’ sentiments toward robotic surgery by using data on these sentiments available from social media platforms, such as X (formerly Twitter). Health care professionals increasingly use social media to share insights, experiences, and opinions, contributing to a broader conversation about medical practices and innovations. Posts shared by physicians on social media offer insights into their perspectives, providing a unique setting for evaluating discussions on robotic surgery through sentiment analysis.

We focused on robotic surgery because it is a highly debated topic in the surgical field, particularly regarding its cost effectiveness, long-term benefits, and potential conflicts of interest due to industry payments to physicians.^6^ Previous studies have delved into the link between industry payments, physician specialty, and physician demographics, showing that male physicians and those in surgical subspecialities were more likely to receive higher-value industry-related payments.^7,8,9^ These payments cover various purposes, including education and consulting (see eTable 1 in Supplement 1 for other payment categories), and appear to have the ability to influence physicians; for instance, one study found that patients are more likely to receive a medical device made by the manufacturer that gave the largest payments to physicians.^10^ In addition, physicians publicly endorse on social media the drugs and devices made by the manufacturers from whom they have accepted payments, sometimes without disclosing compensation.^11,12^ Research also indicates that the da Vinci Surgical System accounts for the highest industry payments to physicians compared with other medical devices.^13^

Despite the significant industry influence and social media activity surrounding robotic surgery, to our knowledge, previous studies have not specifically analyzed physician sentiments on social media toward this technology in the context of industry payments. Given the substantial industry investments and ongoing discourse regarding the effectiveness of robotic technology, our study aims to objectively capture and formally document physicians’ sentiments toward robotic surgery on social media platforms while examining the potential association of these sentiments with industry payments. This approach addresses a gap in the existing literature by providing new insights into how industry relationships may influence public discourse among physicians.

## Methods

### Study Design and Setting

This cohort study had 3 objectives. First, we aimed to identify physicians’ sentiments regarding robotic surgery by analyzing posts shared on X. Second, we sought to assess whether sentiments differed between physicians who received payments from Intuitive Surgical Inc, a biotechnology company that manufactures robotic products for use in surgery, and those who did not. Third, we examined changes in sentiments of physicians after receiving payments and whether these changes differed by the amount of payment they received. We analyzed publicly available posts by physicians between March 19, 2009, and April 1, 2024. The protocol was reviewed, and the University of Michigan institutional review board approved the study with a waiver of informed consent because all data used in this study are publicly available information. This study followed the Strengthening the Reporting of Observational Studies in Epidemiology (STROBE) reporting guideline.

### Selection of Participants and Outcomes

We extracted posts from 268 US-based physicians, selected from followers of the Society of American Gastrointestinal and Endoscopic Surgeon (@sages_updates). The analysis focused on posts containing robotics-related key words (eTable 2 in Supplement 1) along with their replies to these posts. We excluded trainees and individuals for whom we could not validate their clinical practices through a review of professional descriptions, geographic locations, and National Provider Identifier (NPI). We used the NPI Registry to ensure physicians were classified under general surgery and surgical subspecialties (Table 1). Payments for these physicians with validated NPIs were identified using the Open Payments website, with the first payment date set as the index date. Of the 268 physicians, 177 (66.0%) received payments, whereas 91 (34.0%) did not. Of the 177 physicians who received payments, 32 had posts shared before and after the first payment date. We further split these 32 physicians into top 25% and bottom 75% payment quantiles to test whether there were differences in changes of sentiment scores between these 2 groups.

**Table 1.** Distribution of Physicians by Specialty

Specialty	Physicians, No. (%) (N = 268)
General surgery	
Surgery	113 (42.2)
Surgical subspecialties	
Colon and rectal surgery	33 (12.3)
Surgical oncology	21 (7.8)
Thoracic surgery (cardiothoracic vascular surgery)	15 (5.6)
Transplant surgery	14 (5.2)
Pediatric surgery	12 (4.5)
Trauma surgery	10 (3.7)
Others	50 (18.7)

We applied a robust sentiment analysis to 4362 posts using the TextBlob library in Python, version 3.12.7 (Python Software Foundation) (eMethods in Supplement 1) to quantify sentiments expressed in text. This method has been previously applied and widely accepted in social science and health care studies,^14,15,16,17,18^ and to our knowledge, it is a novel application to surgical technology. The analysis had 2 outcomes. The first outcome was subjectivity, which was scored from 0 to 1, with higher scores reflecting more subjective opinions and lower scores reflecting objective facts. The second outcome was polarity, ranging from −1 to 1, with higher scores reflecting more positive sentiment, lower scores reflecting more negative sentiment, and 0 indicating neutrality. Examples of sentences with polarity and subjectivity scores are listed in Table 2.

**Table 2.** Examples of Texts With Polarity and Subjectivity Scores^a^

Text	Polarity score	Subjectivity score
Robotic sleeving with our awesome, soon to graduate, bariatric fellow today. Sometimes you get lucky and they are good surgeons and good people. @anonymous you will be missed! @anonymous is lucky to get you. #bariatricsurgery #roboticsurgery #MedTwitter	0.524	0.678
Robotic excision of an interaortocaval suprarenal paraganglioma. Tough spot!	−0.229	0.411
Depends on training/experience of the surgeon/hospital. Robot data that doesn’t take learning curve into account is worthless.	−0.800	0.900
Looking forward to presenting robotic-assisted esophagectomy with the da Vinci Xi System	0	0

^a^ Sentiment scores were calculated using the TextBlob library in Python, which quantifies the polarity and subjectivity of text.

We conducted preprocessing tasks, including converting texts to lowercase and removing punctuation, numerical values, and noninformative text elements. We also conducted lemmatization using the Natural Language Toolkit to convert words to their base forms, ensuring they are accurately reduced to their root forms based on their contextual use in a sentence. Post scraping and sentiment analysis were conducted using Python.

### Statistical Analysis

We conducted a 2-sided *t* test to compare mean sentiment scores between physicians who received payments and those who did not, with the null hypothesis being that there would be no significant difference in mean scores between the 2 groups. For the subset of physicians with posts before and after receiving payments, we used boxplots to visualize sentiment trends. We further stratified the payment group into the bottom 75% and top 25% of payment distribution to explore potential differences in sentiment trends by payment amount.

## Results

Of 268 US-based physicians (154 men [57.5%], 68 women [25.4%], and 46 physicians [17.2%] with sex not reported), 113 (42.2%) specialized in general surgery and 155 (57.8%) were in surgical subspecialties. The surgical subspecialties included colon and rectal surgery (33 [12.3%]), surgical oncology (21 [7.8%]), thoracic surgery (15 [5.6%]), transplant surgery (14 [5.2%]), pediatric surgery (12 [4.5%]), and trauma surgery (10 [3.7%]) (Table 1). Most physicians practiced in Ohio (30 [11.2%]), California (26 [9.7%]), Texas (25 [9.3%]), Florida (23 [8.6%]), New York (23 [8.6%]), and Illinois (16 [6.0%]).

**Figure 1.**
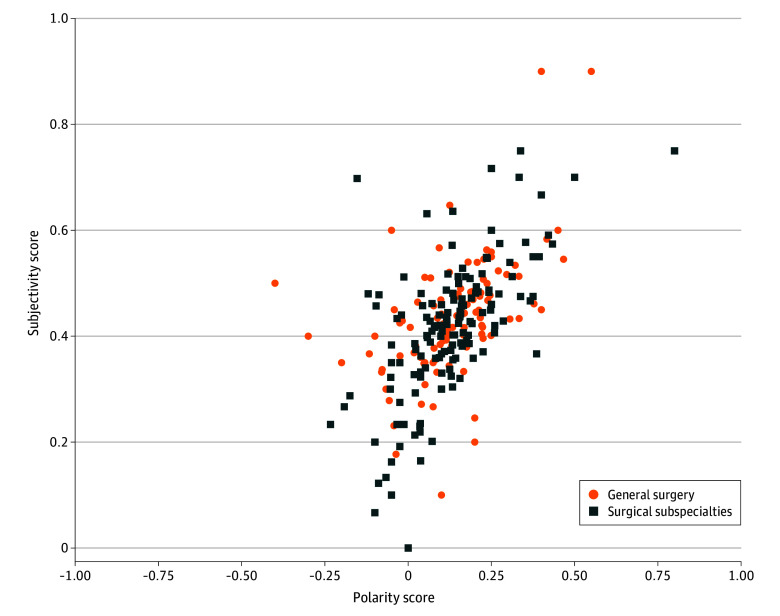
Scatterplot Illustrating the Sentiments of Physicians in Their Posts Containing Robotic-Related Key Words, Comparing Those in General Surgery With Those in Surgical Subspecialties Surgical subspecialties include all specialties listed in Table 1 except for the “surgery” specialty. The x-axis represents the polarity of sentiments, ranging from negative (−1.0) to positive (1.0). The y-axis represents the subjectivity of sentiments, ranging from factual (0.0) to opinion based (1.0). Each point represents an individual physician’s sentiment score derived from their social media posts. Sentiment scores were calculated using the TextBlob library in Python, which quantifies the polarity and subjectivity of text.

Based on 4362 social media posts, the overall sentiment scores for most physicians ranged from −0.25 to 0.5 (mean [SD] score, 0.1 [0.2]; 95% CI, 0.09-0.13), as shown in Figure 1, reflecting generally positive views on robotic surgery. There were no significant differences in views on robotic surgery between physicians in general surgery (mean [SD] score, 0.1 [0.2]; 95% CI, 0.08-0.14) and those in surgical subspecialties (mean [SD] score, 0.1 [0.2], 95% CI, 0.09-0.14). Subjectivity scores ranged from 0 to 0.9, with a mean (SD) subjectivity score of 0.4 (0.2) (95% CI, 0.38-0.41), suggesting the posts were relatively objective but also interspersed with personal insights.

**Figure 2.**
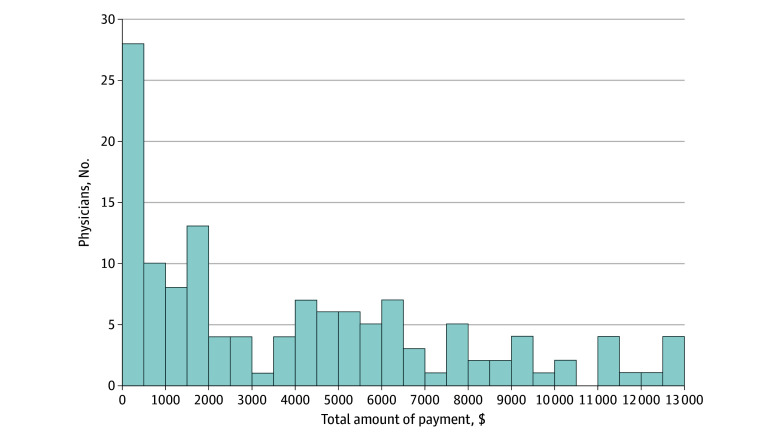
Distribution of Total Industry Payments Received by Physicians in the Bottom 75% of Payment Amounts The total number of physicians in the bottom 75% was 133. The x-axis represents the total amount of payment in US dollars, ranging from $20.68 to $12 896. The y-axis shows the number of physicians within each bin, with each bin representing $500 increments.

Among the 177 physicians who received industry payments, the amounts ranged from $20.68 to $1.2 million. Most of the 177 physicians (133 [75.1%]) received no more than $12 896 in total industry payments from (Figure 2). There were no significant differences in mean polarity scores between physicians who received payments and those who did not (mean [SD] score, 0.12 [0.2]; 95% CI, 0.09-0.14 vs 0.1 [0.2]; 95% CI, 0.07-0.14), suggesting that physicians’ posts were relatively positive on average, regardless of payment status.

**Figure 3.**
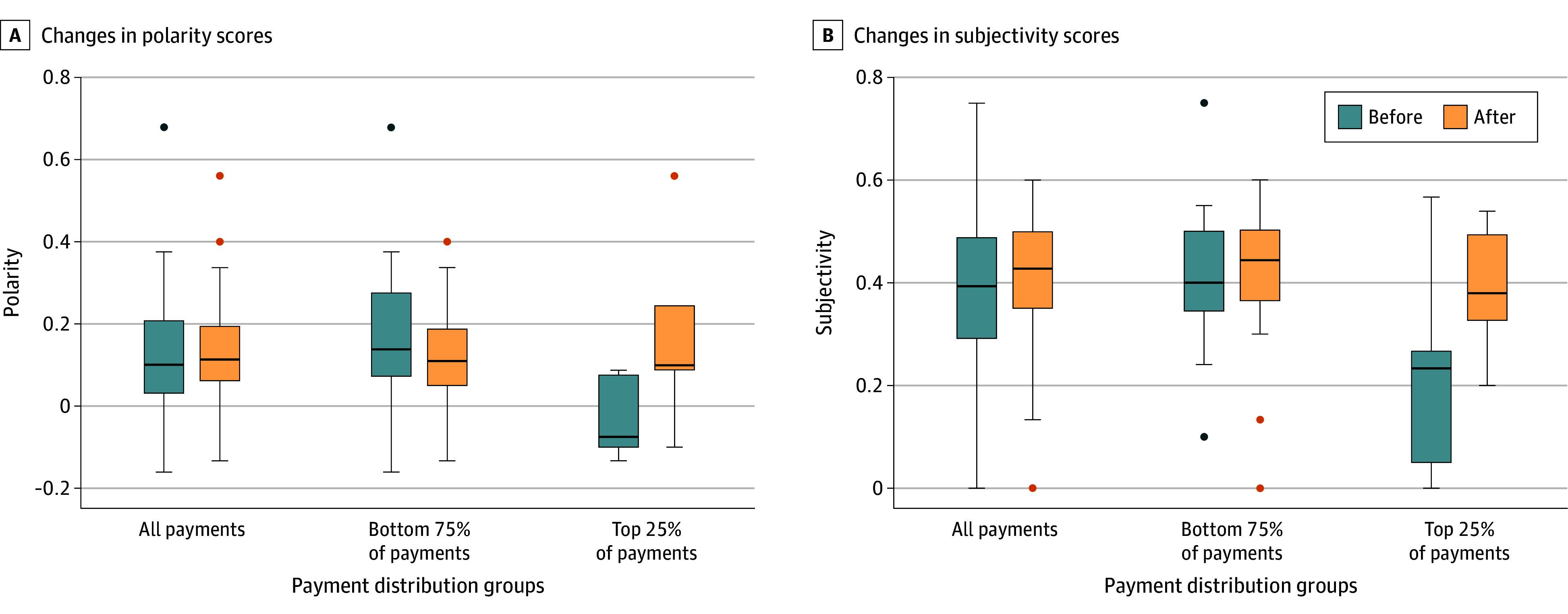
Changes in Polarity and Subjectivity Scores Before and After Receiving Industry Payments These boxplots illustrate the changes in polarity and subjectivity scores of physicians’ posts on X (formerly Twitter) before and after receiving industry payments from Intuitive Surgical Inc. The charts have 3 groups: all physicians (n = 32), physicians in the bottom 75% of payment amounts (n = 24), and physicians in the top 25% of payment amounts (n = 8). The numbers in each boxplot represent median scores. The circles represent outliers both before and after receiving industry payments.

For all 32 physicians who received payments and posted before and after receiving industry payments, there was a narrower distribution of positive sentiments after payment. Figure 3A shows that the range of polarity scores became more concentrated after the payment event for these physicians, with a median polarity score of 0.1 (IQR, 0.03-0.21) before payment and 0.1 (IQR, 0.06-0.19) after payment. The IQR of these boxplots decreased after payment, indicating fewer extreme values and a tighter distribution.

Among these 32 physicians, those in the bottom 75% of the payment distribution had similar sentiment scores before (median score, 0.14 [IQR, 0.07-0.28]) and after (median score, 0.12 [IQR, 0.05-0.19]) receiving industry payments. This group also demonstrated a tighter distribution of polarity scores after payment, as evidenced by the reduced IQR. Physicians in the top 25% of the payment distribution showed a greater change in sentiments. The median polarity score shifted from −0.08 (IQR, –0.1 to 0.08) before receiving payment to 0.10 (IQR, 0.09-0.24) after receiving payment, indicating a shift toward more positive sentiment.

When comparing physicians’ posts before and after receiving industry payments, their posts had a median subjectivity score of 0.39 (IQR, 0.29-0.49) before the payment event and 0.43 (IQR, 0.35-0.50) after the payment, with a narrower distribution of subjectivity (Figure 3B). The median subjectivity score for physicians at the bottom 75% of the payment distribution increased from 0.40 (IQR, 0.35-0.50) to 0.44 (IQR, 0.36-0.50) after receiving industry payments. Physicians whose industry payments were in the top 25% of the payment distribution showed a larger difference, with a median score of 0.2 (IQR, 0.05-0.27) before receiving industry payment and a median score of 0.4 (IQR, 0.33-0.49) after payment. Their subjectivity score became narrower, showing a more concentrated score above 0.2.

## Discussion

This cohort study examined the social media posts of 268 US-based physicians regarding robotic surgery, focusing on their sentiments and the possible influence of payments from a biotechnology company that manufactures robotic products for use in surgery. Analysis of 4362 posts indicated generally positive sentiments with moderate subjectivity, reflecting a blend of objective and personal insights from the physicians. No significant differences were found in sentiment scores between physicians who received industry payments and those who did not. A more detailed examination of physicians who received industry payments and had posted before and after receiving payments showed a different phenomenon. Physicians in the top 25% of payment distribution had posts with more positive and subjective tones after receiving payments, whereas those in the bottom 75% of the payment distribution did not show much difference in sentiments before and after receiving industry payments. These results suggest that while financial ties with the company were not significantly associated with alterations in overall physician sentiments, receipt of a large amount of industry payments was associated with a difference. In other words, the overall sentiments are uniform before and after receiving industry payments, but the most highly paid physicians expressed more positive and subjective sentiments toward robotic surgery.

Our study demonstrated the potential of sentiment analysis to understand physician perceptions of robotic surgery. It builds on previous research that evaluated public perception of robotic surgery and the role of social media in disseminating public health information.^5,19^ This study highlights the importance of understanding physician perceptions, as professional dialogues significantly shape public opinions. Physicians are often seen as reliable sources of medical information, and their endorsements or critiques can influence public attitudes toward emerging medical technologies. Positive sentiments can foster acceptance of new technologies such as robotic surgery, while negative sentiments can lead to skepticism and distrust. Social media platforms can amplify these influences by enabling physicians to reach a broader audience.

### Limitations

This study has some limitations. Physicians active on X may not be representative of the broader physician community, potentially limiting the generalizability of the findings. However, our study emphasizes the significance of these online dialogues, as they reflect and could influence public and professional opinions. In addition, while scraping key words such as “robotic” captures relevant content, it may not fully account for the nuanced influences of personal experiences with robotic surgery or broader industry trends. However, by comparing sentiments between 2 different groups of physicians, the primary factors behind differences in sentiment can still be associated with whether physicians receive payments. The sample size of physicians used for comparison before and after receiving industry payments was also small. The study findings may not be generalizable to other physicians who received payments but are not active on social media. However, because we are interested specifically in physicians who have professionally established themselves on X and linked their NPI to industry payments, we believe the findings should be specific. Future research should explore the correlation between social media sentiments and actual surgical outcomes, as well as the broader adoption of robotic technologies.

## Conclusions

In this cohort study of US-based physicians, publicly available social media data were used to quantify perceptions of robotic surgery. Our method uniquely involves verifying physicians’ legitimacy through their NPI and connecting their sentiments to industry payments. Understanding these professional sentiments is crucial given the influential role physicians play in shaping public perceptions. This insight can guide efforts to ensure balanced information dissemination and address potential misinformation on social media. As health care continues to evolve with emerging technologies, monitoring and analyzing social media discourse will become increasingly important for regulating the content shared by medical professionals and maintaining the integrity of health information.

## Notes

Supplement 1. **eMethods.** Broader Description of TextBlob **eTable 1.** Nature of Industry Payment Categories **eTable 2.** Robotic-Related Keywords

Supplement 2.
Data Sharing Statement
