# Supplementary material for: Industry Payments and Sentiments Toward Robotic Surgery Among US Physicians
Source: JAMA Netw Open. 2025 Feb 7;8(2):e2458552. doi: 10.1001/jamanetworkopen.2024.58552 (PMC11806388; doi:10.1001/jamanetworkopen.2024.58552)
Supplement: Supplement 1. — eMethods. Broader Description of TextBlob eTable 1. Nature of Industry Payment Categories eTable 2. Robotic-Related Keywords [file jamanetwopen-e2458552-s001.pdf]

Supplementary Online Content

Loh WS, Ibrahim AM, Sheskey S, Stone CM, Sheetz KH. Industry payments and sentiments toward robotic surgery among US physicians. *JAMA Netw Open.* 2025;8(2):e2458552. doi:10.1001/jamanetworkopen.2024.58552

eMethods. Broader Description of TextBlob

eTable 1. Nature of Industry Payment Categories

eTable 2. Robotic-Related Keywords

This supplementary material has been provided by the authors to give readers additional information about their work.

© 2025 Loh WS et al. *JAMA Network Open.*

eMethods. Broader Description of TextBlob

TextBlob, a Python library, is designed for processing textual data. This tool evaluates two main sentiment metrics: polarity and subjectivity. Polarity scores range from -1.0 (strongly negative) to 1.0 (strongly positive), with 0 indicating neutrality. Subjectivity scores span from 0.0 (completely objective) to 1.0 (highly subjective). TextBlob leverages a predefined lexicon to assign sentiment scores at the sentence level. The sentiment of each sentence is determined by analyzing the sentiment scores of the words within it, adjusted according to their contextual use. For broader texts, such as entire sets of posts per physician, the overall sentiment is calculated by averaging sentiment scores of all sentences within these texts.

© 2025 Loh WS et al. *JAMA Network Open.*

eTable 1. Nature of Industry Payment Categories

Nature of Payment
• Education<br>• Travel and Lodging<br>• Compensation for services other than consulting, including serving as faculty or as a speaker at a venue other than a continuing education program<br>• Long term medical supply or device<br>• Food and beverage<br>• Space rental or facility fees (teaching hospital only)<br>• Consulting fee<br>• Gift<br>• Debt forgiveness<br>• Royalty or license

© 2025 Loh WS et al. *JAMA Network Open.*

eTable 2. Robotic-Related Keywords

Keywords
Robotic
Robotics
Roboticsurgery
Robotic-assisted
Robo surgery
Robo Surg
Da Vinci
Davinci
Intuitive surgical

© 2025 Loh WS et al. *JAMA Network Open.*
