# Supplementary material for: Industry Payments and Sentiments Toward Robotic Surgery Among US Physicians
Source: JAMA Netw Open. 2025 Feb 7;8(2):e2458552. doi: 10.1001/jamanetworkopen.2024.58552 (PMC11806388; doi:10.1001/jamanetworkopen.2024.58552)
Supplement: Supplement 2. — Data Sharing Statement [file jamanetwopen-e2458552-s002.pdf]

Data Sharing Statement

Loh. Industry Payments and Sentiments Toward Robotic Surgery Among US Physicians. *JAMA Netw Open*. Published February 07, 2025. doi:10.1001/jamanetworkopen.2024.58552

Data
Data available: No
